# The effect of smoking cessation on work disability risk: a longitudinal study analysing observational data as non-randomized nested pseudo-trials

**DOI:** 10.1093/ije/dyz020

**Published:** 2019-02-27

**Authors:** Jaakko Airaksinen, Jenni Ervasti, Jaana Pentti, Tuula Oksanen, Sakari Suominen, Jussi Vahtera, Marianna Virtanen, Mika Kivimäki

**Affiliations:** 1 Department of Psychology and Logopedics, Medicum, University of Helsinki, Helsinki, Finland; 2 Department of Occupational Health, Finnish Institute of Occupational Health, Helsinki, Finland; 3 Clinicum, University of Helsinki, Helsinki, Finland; 4 Department of Public Health, University of Turku and Turku University Hospital, Turku, Finland; 5 Institute of Public Health and Caring Sciences, University of Uppsala, Uppsala, Sweden; 6 Department of Epidemiology and Public Health, University College London, London, UK

**Keywords:** Smoking, cessation, work disability, pseudo-trial

## Abstract

**Background:**

Smoking increases disability risk, but the extent to which smoking cessation reduces the risk of work disability is unclear. We used non-randomized nested pseudo-trials to estimate the benefits of smoking cessation for preventing work disability.

**Methods:**

We analysed longitudinal data on smoking status and work disability [long-term sickness absence (≥90 days) or disability pension] from two independent prospective cohort studies—the Finnish Public Sector study (FPS) (*n *= 7393) and the Health and Social Support study (HeSSup) (*n* = 2701)—as ‘nested pseudo-trials’. All the 10 094 participants were smokers at Time 1 and free of long-term work disability at Time 2. We compared the work disability risk after Time 2 of the participants who smoked at Time 1 and Time 2 with that of those who quit smoking between these times.

**Results:**

Of the participants in pseudo-trials, 2964 quit smoking between Times 1 and 2. During the mean follow-up of 4.8 to 8.6 years after Time 2, there were 2197 incident cases of work disability across the trials. Quitting smoking was associated with a reduced risk of any work disability [summary hazard ratio = 0.89, 95% confidence interval (CI) 0.81–0.98]. The hazard ratio for the association between quitting smoking and permanent disability pension (928 cases) was of similar magnitude, but less precisely estimated (0.91, 95% CI 0.81–1.02). Among the participants with high scores on the work disability risk score (top third), smoking cessation reduced the risk of disability pension by three percentage points. Among those with a low risk score (bottom third), smoking cessation reduced the risk by half a percentage point.

**Conclusions:**

Our results suggest an approximately 10% hazard reduction of work disability as a result of quitting smoking.

**Key Messages**

- In addition to the overall negative health effects, smoking is also a risk factor for work disability. However, the effect of smoking cessation on the risk of work disability is unclear.
- Using a non-randomized nested pseudo-trial design, we showed that smoking cessation may reduce the hazard of work disability by over 10%, compared with continuing smoking.
- In absolute terms, smokers at a high overall risk of work disability could expect their risk of work disability to fall on quitting by three percentage points, from 35% to 32%.

## Introduction

Smoking is one of the leading causes of preventable death^1^ and disease burden.^2^ As well as being a risk to health in general, it has also been identified as a risk factor for work disability.^3–9^ Although the damage done to health by smoking is long-lasting, smoking cessation nonetheless seems to reduce the risk of adverse health outcomes in the long run.^10^^,^^11^ However, few studies have examined the benefits of smoking cessation for the prevention of work disability.

Two studies using large cohorts of twins from Sweden^12^ and Finland^13^ compared the risk of work disability due to musculoskeletal diagnosis of never-smokers, current smokers, and those who had changed their smoking habits. In the Swedish study, continued smoking was associated with an increased risk of work disability after adjusting for multiple confounders. An increase or decrease in smoking (analysed as change in smoking) was not associated with work disability.^12^ The Finnish study found smoking to be a risk factor for work disability among persistent smokers, and an attenuated risk among those who quit smoking.^13^ However, both studies had their limitations. The Swedish study determined change in smoking behaviour imprecisely, as change within the past 25 years before follow-up; and the Finnish study had relatively few quitters. Also a further Swedish study, which examined only women, found that smokers were up to five percentage points more likely to be granted disability pension than non-smokers, although the effect was greatly attenuated after controlling for familial background.^14^

To quantify the potential benefits of smoking cessation in terms of reducing the risk of work disability, we analysed longitudinal data from two large prospective cohort studies as non-randomized nested pseudo-trials.^15^ More specifically, we included only those who were current smokers at baseline (Time 1), identified those who reported having given up smoking at Time 2, and then followed both the quitters and the persistent smokers via linkage to electronic records of national health registers for work disability for an average of 8 to 9 years.

## Methods

### Study design and participants

Participants were drawn from the Finnish Public Sector study (FPS)^16^ and the Health and Social Support study (HeSSup).^17^ They were eligible for the present analysis if they had responded to two successive surveys, labelled as Time 1 (T1) and Time 2 (T2); smoked at T1; had information regarding their smoking status at T2; and were alive and not on long-term sickness absence, retired or on disability pension at the start of the follow-up for long-term sickness absence and disability pension after T2.

The FPS surveys took place in 1997–98 (*n *= 16 948, response rate 70%), 2000–02 (*n* = 48 598, response rate 68%), 2004 (*n *= 48 076, response rate 66%) and 2008 (*n* = 52 891, response rate 71%). Altogether 7393 participants from FPS were eligible for the study. The four surveys were used to conduct three non-randomized nested ‘trials’, that is from 1997 to 2000–02, 2000–02 to 2004 and 2004 to 2008. As participants could be included in several trials, we had altogether 10 404 observations for FPS. In HeSSup, 11 886 (76%) of the respondents to the survey in 1998 (T1) took part in the 2003 (T2) survey and of these, 2701 smokers at T1 were eligible for the current study. This study allowed us to conduct one non-randomized trial.

We linked the participants to electronic records on work disability, sickness absences, statutory retirement and mortality, which were available until the end of 2011 for FPS and until the end of 2013 for HeSSup. We conducted the linkage using the unique personal identification numbers assigned to all Finnish citizens. Follow-up was from the beginning of the following year of T2 for both quitters and those who remained smokers. For all participants, follow-up was until long-term sickness absence, disability pension or old age pension, death or end of follow-up, whichever came first.

### Ascertaining work disability

Work disability was defined if the participant had a long-term sickness absence (≥90 days) or was granted a disability pension. The first is denoted as ‘any work disability’, because full disability pension can be granted after having received sickness allowance for 300 working days, and in both cohorts all disability pensions were preceded by long-term sickness absences, effectively making disability pensions special cases among the long-term sickness absence cases. Both studies obtained sickness absence records from the Social Insurance Institution of Finland, which keeps national registers of long-term sickness absences. We obtained records of granted work disability pensions, including starting date and type of pension, from the Finnish Centre of Pensions.

### Smoking measurements and baseline characteristics

Smoking status was self reported and dichotomized (0 = non-smoker/former smoker, 1 = current smoker) at T1 and T2. Sociodemographic factors at T1, treated as covariates, included age, sex and socioeconomic status. In FPS, dichotomized socioeconomic status (SES, low vs high) was obtained from register-based occupation class, and in HeSSup from self-reported educational level. The low SES group included manual workers such as construction, manufacturing and transportation workers (FPS); those with only vocational school education; and those who had attended a vocational course, had apprenticeship training or had no vocational education (HeSSup). High SES included participants who worked as administrators, managers, experts, specialists, office workers, customer service workers, sales workers and hospital nurses in FPS, and participants with university, polytechnic or college-level education in HeSSup.

Other T1 covariates included body mass index (BMI), physical activity and alcohol consumption. BMI was calculated from self-reported height and weight (weight in kg/height in m^2^) and dichotomized into obese (BMI  ≥ 30) and non-obese (BMI <30). Physical activity was measured as self-reported weekly hours of physical activity of different intensities: walking, brisk walking, jogging and running. Participants reporting less than 30 min of brisk walking, jogging or running per week were categorized as inactive, and active if more than this. Further, those who reported only walking were categorized as inactive.^18^ Alcohol consumption was measured as self-reported weekly consumption of beer, wine and spirits, which was transformed into units of alcohol per week. We also included physician-diagnosed chronic diseases at T1 as covariates. The self-reported diseases elicited in the survey were matched with the diseases from the Global Burden of Disease Study, which contribute to global disability-adjusted life years:^19^ asthma, myocardial infarction, angina pectoris, cerebrovascular diseases, migraine, depression and diabetes.

### Statistical analysis

As our data were from observational cohort studies, we used counterfactual modelling with a non-randomized nested pseudo-trial design in our analysis.^15^ We divided the participants into ‘treatment’ (i.e. quit smoking after T1) and ‘reference’ (i.e. continued smoking) groups according to their smoking status at T2. We examined the differences between baseline covariates at T1 of participants who quit smoking between T1 and T2 and those who did not, using χ^2^ and t tests as appropriate.

For the main analysis, we used Cox proportional hazard models to compare the risk of work disability among those who quit smoking between T1 and T2 (the ‘treatment’ group) with that of those who smoked at T1 and T2 (the ‘reference’ group), with hazard ratios (HR) and their 95% confidence intervals (CI). As we had data at four different time points for the FPS participants, we conducted three separate non-randomized trials.^15^ We then pooled together these trials. As some participants took part in several trials, we used a robust variance estimator to account for within-person correlation, which would otherwise have resulted in incorrect confidence intervals. For HeSSup we could only conduct one trial. We separately analysed associations with any work disability (long-term sickness absence or disability pension) and disability pension only. At the start of follow-up, we adjusted the analyses for age (categorized to <35, 35–39, 40–44, 45–49, 50–54 and >55), sex, socioeconomic status (SES), BMI, physical activity, alcohol consumption and chronic diseases. The number of participants in each analysis varied slightly due to missing values in the covariates. Study-specific estimates were combined using fixed-effect meta-analysis.

To account for the fact that some participants took up smoking again after cessation, we computed adherence-adjusted estimates for the effect of cessation on work disability.^15^ Only participants from FPS who took part in three subsequent surveys (T1, T2 and T3), and whose smoking status remained the same from T2 to T3, were included in these analyses, which were conducted in the same manner as the main analysis. We also examined whether the effect of smoking cessation on the risk of work disability was modified by follow-up period, age, sex, socioeconomic position, obesity or physical activity. Of these, we examined the effect modification of the follow-up period using a logarithm of the time.

To examine the potential benefits of quitting smoking at different levels of estimated disability risk, we determined the participants’ 10-year risk of work disability at T1 (the time all participants smoked) using a recently developed eight-item work disability risk score.^9^ For each participant, this score was calculated using T1 data on age, self-rated health, number of sickness absences during the previous year, SES, number of chronic illnesses, difficulty falling asleep, BMI and smoking.^9^ We divided the participants into three equal-sized groups according to their risk score at the time they were all still smoking, and for each group computed the difference between work disability risk from continued smoking and smoking cessation.^9^

## Results

Table 1 shows the descriptive characteristics for the participants of FPS and HeSSup at T1, stratified by smoking status at T2**.** In both cohort studies, those who quit smoking had higher SES, were younger and consumed less alcohol than those who continued smoking. In FPS, they were also more physically active and in HeSSup they were more often not obese. There was no sex difference between the groups of quitters and continued smokers in either cohort.

**Table 1.** Descriptive statistics for study participants smoking at Time 1. Frequency (percentage), unless otherwise stated

	FPS (*n *= 7395; 10 404 observations)			HeSSup (*n* = 2701)		
	Smoking at Time 2	Quit smoking by Time 2		Smoking at Time 2	Quit smoking by Time 2	
	(*n* = 8116)	(*n* = 2288)	*P* for difference	(*n* = 2025)	(*n* = 676)	*P* for difference
Age, mean (SD)	44.3 (8.1)	43.3 (8.7)	<0.01	36.0 (10.4)	33.5 (10.6)	<0.01
Sickness absence during follow-up	1389 (17)	293 (13)	<0.01	408 (20)	107 (1)	0.02
Disability pension during follow-up	620 (8)	118 (5)	<0.01	155 (8)	35 (5)	0.04
Year of follow-up, mean (SD)						
Sickness absence	5.2 (2.7)	4.8 (2.5)	<0.01	8.3 (2.9)	8.6 (2.6)	<0.01
Disability pension	5.6 (2.9)	5.1 (2.4)	<0.01	8.9 (2.2)	9.2 (1.9)	<0.01
Sex						
Men	1925 (24)	569 (25)	0.27	841 (42)	291 (43)	0.52
Women	6191 (76)	1719 (75)		1184 (58)	385 (57)	
Socioeconomic position						
Low SES	2082 (26)	451 (20)	<0.01	1271 (63)	383 (57)	<0.01
High SES	6011 (74)	1832 (80)		736 (36)	289 (43)	
Obesity (BMI >30kg/m^2^)						
Not obese	6948 (86)	1979 (86)	0.28	1817 (90)	626 (93)	0.02
Obese	1003 (12)	263 (11)		196 (10)	44 (7)	
Mean alcohol consumption, units per week (IQR)	8.0 (2-10)	7.1 (2-9)	<0.01	8.6 (2-11)	7.4 (2-11)	<0.01
Physical activity						
Active	6001 (74)	1757 (77)	0.01	1512 (75)	520 (77)	0.35
Inactive	2057 (25)	517 (23)		497 (25)	154 (23)	
Asthma						
No	7396 (91)	2088 (91)	0.71	1916 (95)	646 (96)	0.52
Yes	452 (6)	122 (5)		101 (5)	29 (4)	
Myocardial infarction						
No	7819 (96)	2199 (96)	0.51	2003 (99)	673 (100)	0.85
Yes	29 (0)	11 (0)		9 (0)	2 (0)	
Angina pectoris						
No	7783 (96)	2187 (96)	0.41	1983 (98)	673 (100)	0.03
Yes	65 (1)	23 (1)		28 (1)	2 (0)	
Cerebrovascular diseases						
No	7748 (95)	2190 (96)	0.19	1990 (98)	669 (99)	0.82
Yes	100 (1)	20 (1)		22 (1)	6 (1)	
Migraine						
No	6344 (78)	1752 (77)	0.11	1620 (80)	568 (84)	0.04
Yes	1504 (19)	458 (20)		397 (20)	108 (16)	
Depression						
No	6712 (83)	1927 (84)	0.05	247 (12)	70 (10)	0.22
Yes	1136 (14)	283 (12)		7 (0)	1 (0)	
Diabetes						
No	7664 (94)	2162 (94)	0.69	46 (2)	6 (1)	0.03
Yes	184 (2)	48 (2)		9 (0)	1 (0)	

SD, standard deviation; IQR, interquartile range.

Supplementary Table 1 (available as Supplementary data at *IJE* online) shows the results of the analysis of baseline covariates as predictors of any work disability and of disability pension only. Being older was associated with an increased risk of both work disability outcomes in both study cohorts. Further, in the FPS cohort, female sex and lower socioeconomic status were associated with an increased risk of both outcomes, and being obese and physically inactive with an increased risk of long-term sickness absence. In the HeSSup cohort, obesity and physical inactivity together with being female were associated with an increased risk of long-term sickness absence.

The pseudo-trials of FPS found 1682 (16.2%) incident cases of long-term sickness absence (≥90 days) during a mean follow-up of 5.1 years, and 738 (7.1%) disability pensions were granted during a mean follow-up of 5.5 years. In HeSSup, 515 (19.1%) participants had a long-term sickness absence (mean follow-up 8.4 years), and 190 (7.0%) were granted disability pension (mean follow-up 9.0 years). Table 2 shows the main diagnosis groups for which any work disability was granted in both cohorts. The three most common causes of any work disability were: diseases of the musculoskeletal system and connective tissue; mental and behavioural disorders; and injury, poisoning and certain other consequences of external causes. In both cohort studies, quitters were twice more likely to be granted any work disability on the basis of respiratory diseases than smokers.

**Table 2.** Common reasons for granting any work disability by main ICD-10 diagnosis group in both study cohorts, and percentages of all disability pensions by row

		C	D	F	G	I	J	K	L	M	S
FPS	Smokers	118 (8)	14 (1)	235 (17)	60 (4)	96 (7)	15 (1)	20 (1)	12 (1)	640 (46)	140 (10)
	Quitters	19 (6)	2 (1)	45 (15)	12 (4)	16 (5)	7 (2)	8 (3)	0 (0)	147 (50)	26 (9)
HeSSup	Smokers	27 (6)	1 (0)	73 (17)	18 (4)	18 (4)	9 (2)	7 (2)	5 (1)	175 (42)	56 (13)
	Quitters	8 (7)	0 (0)	20 (19)	2 (2)	9 (8)	4 (4)	1 (1)	1 (1)	42 (39)	13 (12)

C, neoplasms; D, diseases of the blood and blood-forming organs and certain disorders involving the immune mechanisms; F, mental and behavioural disorders; G, diseases of the nervous system; I, diseases of the circulatory system; J, diseases of the respiratory system; K, diseases of the digestive system; L, diseases of the skin and subcutaneous tissue; M, diseases of the musculoskeletal system and connective tissue; S, injury, poisoning and certain other consequences of external causes.

**Figure 1.**
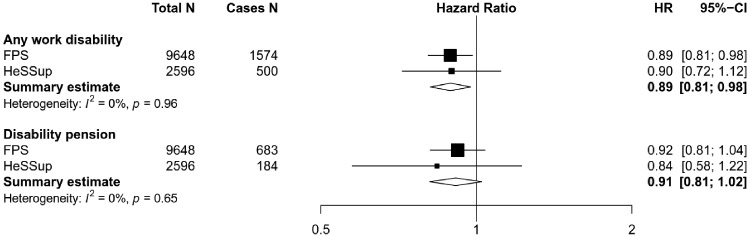
Cox regression analysis on the association between quitting smoking and the risk for work disability. Study specific and pooled hazard ratios are adjusted for age, sex, socioeconomic status, obesity, physical activity, alcohol consumption, and chronic diseases.

Figure 1 shows the association of smoking cessation, compared with continued smoking, with any work disability and with disability pension only. In a meta-analysis of the estimates from the two cohort studies, the age, sex and SES-adjusted HR for quitting smoking and any work disability was 0.88 (95% CI 0.79–0.99). This association remained in the multivariable adjusted model after further adjustment for obesity, physical activity, alcohol consumption and chronic diseases; HR 0.89 (95% CI 0.81–0.98). The HR for the association between smoking cessation and the risk of disability pension was of a similar magnitude, but less precisely estimated (age, sex and SES-adjusted HR 0.87, 95% CI 0.73–1.02 and multivariable-adjusted HR 0.91, 95% CI 0.81–1.02). In the study-specific analyses, the point estimates for disability pension HRs differed somewhat, although the confidence intervals overlapped completely and we observed no heterogeneity between the studies. Supplementary Table 2 (available as Supplementary data at *IJE* online) provides more detailed results.

The smoking behaviour of 3480 participants did not change during follow-up from T2 to T3 (a third survey) in the FPS cohort. Using the FPS cohort, the adherence-adjusted HR for quitting smoking and any work disability was 0.90 (95% CI 0.83–0.99). The adherence-adjusted HR for quitting smoking and the reduced risk of disability pension was of the same magnitude but less precisely estimated: HR = 0.93 (95% CI 0.76–1.15). The analysis of effect modifiers (Supplementary Table 3, available as Supplementary data at *IJE* online) showed that only physical inactivity and follow-up time acted as effect modifiers. Being physically inactive when quitting smoking lowered the risk of any work disability, and similarly, longer follow-up times attenuated the protective effect of quitting on any work disability.

**Figure 2.**
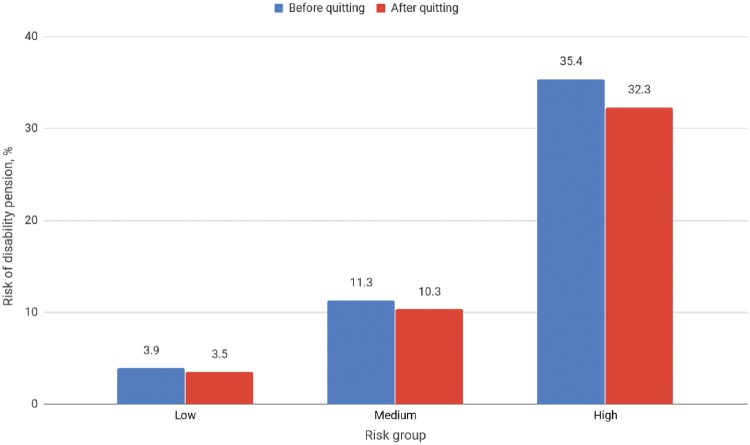
Mean absolute 10-year risks for disability pension before and after quitting smoking in three risk groups. Participants were divided into three equal-size groups according to their 8-item work disability score when smoking (score range 0.1% to <6.5% for low risk group, 6.5% to <18.3% for medium risk group, and 18.3% or higher for high risk group).

Figure 2 illustrates the mean estimated 10-year risk reduction of disability pension when quitting smoking. The participants were divided into three equal-sized groups according to their eight-item disability risk score at the time when they all smoked (score range 0.1% to <6.5% for the low-risk group, 6.5% to <18.3% for the medium-risk group, and 18.3% or higher for the high-risk group). The mean risk reduction for all participants was 1.5% (from 16.9% to 15.4%), suggesting that approximately 15 cases of work disability would be prevented in a group of 1000 smokers who quit smoking. For the participants in the highest third of overall work disability risk, the mean risk of disability pension decreased from 35.4% (not quitting) to 32.3% (quitting), whereas the participants in the lowest third of overall disability risk saw a mean risk reduction from 3.9% (not quitting) to 3.5% (quitting). Thus, for every 1000 smokers who quit smoking in the high-risk group, 32 cases of disability pension would be prevented. For the low-risk group, the number of prevented cases of disability pension would be four.

## Discussion

We used a non-randomized nested pseudo-trial study design to estimate the effect of smoking cessation on the risk of work disability. Pooled estimates from the two study cohorts suggest that smoking cessation may lower the HR of any work disability (sickness absences lasting ≥90 days or disability pension) by 11%. The corresponding reduction in the hazard ratio for disability pension was 9%. The estimates of risk reduction from the adherence-adjusted analysis were in line with the main results, although the risk reduction estimate for disability pension was uncertain due to the smaller number of participants in these analyses. According to the analyses stratified by the baseline overall disability risk of theeight8-item risk score,^9^ people in the high-risk group who quit smoking could expect to see their risk of disability pensions falling by three percentage points during the next 10 years, from 35.4% to 32.3%. The corresponding risk reduction as a result of quitting smoking is one percentage point (from 11.3% to 10.3 %) in a population with an intermediate risk, and less than half a percentage point (from 3.9% to 3.5 %) among low-risk individuals.

Previous research on the effect of smoking cessation on the risk of work disability has been scarce. An earlier study using a large Finnish twin cohort^13^ examined how quitting smoking affected the risk of work disability due to a musculoskeletal illness diagnosis. The findings suggested that quitting smoking might mitigate some of the risk of disability pension. Our findings expand on this evidence by providing a quantitative estimate for risk reduction in relation to disability pension.

The previously developed and validated eight-item risk prediction tool provides occupational health professionals and workplaces with a scaleable way to estimate employees’ 10-year absolute risk of work disability.^9^ For high-unit-cost prevention, or when resources for prevention are limited, workplaces might prefer to target smoking cessation interventions only towards smokers with the highest overall risk of work disability.^20^ In this study, the estimated three percentage point risk reduction among smokers in the top third of the overall work disability risk score translates to 32 prevented cases of disability pension for every 1000 smokers who quit smoking. This illustrates the expected benefits of quitting smoking compared with continuing smoking in a high-risk population. The corresponding risk reduction was 1.5 percentage points among all smokers in this study, suggesting that approximately 15 cases would be prevented per every 1000 quitters in such a population of smokers. This provides an estimate of the potential benefits of a successful population-wide prevention strategy. Obviously, these estimates may be dependent on the characteristics of the working population and only relate to successful intervention cases.

The main strength of this study is its use of a non-randomized nested pseudo-trial study design to emulate the design of a randomized trial in two independent cohort studies. Non-randomized pseudo-trials estimated the effect of a change in the exposure variable (increase or reduction) between two time points before the change in the outcome. This study design is subject to confounding and reverse causation, but probably to a lesser extent than traditional prospective studies with a single baseline assessment and follow-up of the outcome.

However, the design also has limitations. The control group—here, smokers—and treatment group—quitters—at Time 2 may not have been fully comparable at the beginning of follow-up, as participants were not randomly assigned to these groups. Even after controlling for various sociodemographic and lifestyle factors and chronic diseases, some variables may have remained unmeasured, such as the intensity and duration of smoking or the different reasons for quitting smoking. This may confound the associations between cessation and the risk of work disability. Likewise, as has previously been suggested, familial background may account for a portion of the association.^14^

## Conclusions

We conducted non-randomized nested pseudo-trials to complement evidence from traditional epidemiological studies of observational data. Using two large independent longitudinal cohorts, we estimated that smoking cessation is likely to lower the hazard ratio for long-term sickness absence by 11%, compared with continued smoking. In absolute terms, the benefits of quitting smoking seem to be greater in populations with a high overall risk of work disability. Ideally, these findings should be confirmed by future randomized controlled trials on smoking cessation.

## Funding

J.A. was supported by the Finnish Work Environment Fund (No. 115421). M.K. was supported by NordForsk, the Medical Research Council (K013351, S011676, MR/R/024227/1), NordForsk, Academy of Finland (311492) and Helsinki Institute of Life Science Fellowship.

**Conflict of interest:** None declared. 

## Supplementary Material

Supplementary Data
