# Supplementary material for: The effect of smoking cessation on work disability risk: a longitudinal study analysing observational data as non-randomized nested pseudo-trials
Source: Int J Epidemiol. 2019 Feb 27;48(2):415–22. doi: 10.1093/ije/dyz020 (PMC6469311; doi:10.1093/ije/dyz020)
Supplement: Supplementary Data [file dyz020_supplementary_data.pdf]

Supplementary Table 1. Hazard ratios of work disability outcomes for covariates from Cox regression models

		Sickness absence (90 days) or disability pension						Disability pension					
		FPS			HeSSup			FPS			HeSSup		
		Cases (%)	HR	95% CI	Cases (%)	HR	95% CI	Cases (%)	HR	95% CI	Cases (%)	HR	95% CI
Age a													
	<35	59 (7)	1	-	6 (1)	1	-	80 (11)	1	-	12 (2)	1	-
	35-39	96 (9)	1.29	(1.01, 1.66)	20 (2)	2.63	(1.63, 4.25)	77 (14)	1.3	(0.95, 1.78)	16 (3)	1.78	(0.84, 3.76)
	40-44	236 (14)	2.11	(1.63, 2.73)	60 (4)	5.08	(2.6, 9.95)	33 (23)	2.3	(1.53, 3.45)	8 (6)	3.54	(1.45, 8.65)
	45-49	363 (17)	2.64	(1.89, 3.67)	141 (7)	9.83	(4.69, 20.6)	167 (27)	2.78	(2.13, 3.63)	73 (12)	7.74	(4.2, 14.25)
	50-54	484 (21)	3.5	(2.8, 4.38)	264 (11)	19.19	(12.57, 29.28)	56 (33)	3.56	(2.53, 5.01)	23 (14)	9.14	(4.55, 18.37)
	55+	444 (19)	4.63	(3.7, 5.8)	247 (11)	30.28	(15.61, 58.73)	102 (24)	4.23	(3.14, 5.7)	58 (14)	16.98	(9.04, 31.89)
Sex b													
	Male	361 (15)	1	-	146 (6)	1	-	184 (16)	1	-	73 (7)	1	-
	Female	1321 (17)	1.15	(1.12, 1.19)	592 (8)	1.3	(1.27, 1.34)	331 (21)	1.33	(1.11, 1.59)	117 (8)	1.15	(0.86, 1.54)
Socioeconomic position c													
	Low	594 (24)	1	-	290 (12)	1	-	362 (22)	1	-	133 (8)	1	-
	High	1083 (14)	1.15	(1.11, 1.19)	445 (6)	1.3	(1.26, 1.33)	151 (15)	1.33	(1.11, 1.59)	56 (6)	1.14	(0.85, 1.53)
Obesity (BMI>30) c													
	No	1424 (16)	1	-	612 (7)	1	-	461 (19)	1	-	166 (7)	1	-
	Yes	220 (18)	1.19	(1.04, 1.36)	115 (9)	1.48	(1.3, 1.69)	52 (22)	1.03	(0.77, 1.38)	23 (10)	1.15	(0.74, 1.79)
Alcohol consumption c													
		na	1	(1, 1.01)	na	1	(0.99, 1)	na	1	(0.99, 1.01)	na	1	(0.99, 1.02)
Physical activity c													
	Active	1187 (16)	1	-	490 (6)	1	-	371 (18)	1	-	135 (7)	1	-
	Inactive	474 (19)	1.24	(1.22, 1.27)	239 (10)	1.47	(1.36, 1.6)	142 (22)	1.2	(0.98, 1.45)	54 (8)	1.14	(0.83, 1.56)
Asthma c													
	No	1489 (16)	1	-	630 (7)	1	-	480 (19)	1	-	178 (7)	1	-
	Yes	128 (23)	1.55	(1.48, 1.63)	73 (13)	2.1	(1.88, 2.34)	32 (25)	1.49	(1.04, 2.13)	12 (9)	1.49	(0.83, 2.69)
Myocardical infacrtion c													
	No	1603 (16)	1	-	693 (7)	1	-	505 (19)	1	-	184 (7)	1	-
	Yes	14 (37)	2.63	(1.7, 4.05)	10 (26)	3.63	(3.33, 3.95)	2004 (100)	1.71	(0.64, 4.59)	2004 (100)	3.61	(1.33, 9.81)
Angina pectoris c													
	No	1589 (16)	1	-	684 (7)	1	-	499 (19)	1	-	185 (7)	1	-
	Yes	28 (34)	2.09	(1.9, 2.3)	19 (23)	2.81	(2.46, 3.21)	10 (33)	1.65	(0.88, 3.1)	4 (13)	1.43	(0.53, 3.87)
Cerebrovascular diseases c													
	No	1587 (16)	1	-	682 (7)	1	-	499 (19)	1	-	182 (7)	1	-
	Yes	30 (27)	1.73	(1.4, 2.13)	21 (19)	2.68	(2.04, 3.53)	10 (37)	2.06	(1.1, 3.87)	5 (19)	2.12	(0.87, 5.19)
Migraine c													
	No	1261 (16)	1	-	546 (7)	1	-	386 (18)	1	-	144 (7)	1	-
	Yes	356 (19)	1.25	(1.17, 1.33)	157 (8)	1.28	(1.18, 1.38)	128 (26)	1.42	(1.15, 1.74)	46 (9)	1.33	(0.95, 1.88)
Depression c													
	No	1266 (15)	1	-	524 (6)	1	-	426 (18)	1	-	148 (6)	1	-
	Yes	351 (26)	1.85	(1.71, 2.01)	179 (13)	2.13	(1.89, 2.41)	85 (27)	1.47	(1.17, 1.87)	41 (13)	1.91	(1.35, 2.71)
Diabetes c													
	No	1557 (16)	1	-	672 (7)	1	-	490 (19)	1	-	177 (7)	1	-
	Yes	60 (27)	1.87	(1.56, 2.25)	31 (14)	2.08	(1.73, 2.5)	22 (43)	2.41	(1.57, 3.71)	13 (25)	3.31	(1.87, 5.85)

a – adjusted for sex, b- adjusted for age, c- adjusted for age and sex

Supplementary Table 2. Detailed study specific results for the association between smoking cessation and any work disability, and disability pension

	Any work disability				Disabilty pension			
	FPS		HeSSup		FPS		HeSSup	
Age 35-39	1.17	(0.93, 1.47)	1.39	(1.01, 1.92)	2.33	(1.55, 3.50)	1.79	(0.84, 3.81)
Age 40-44	1.89	(1.48, 2.42)	2.20	(1.45, 3.35)	4.36	(2.42, 7.85)	2.92	(1.14, 7.43)
Age 45-49	2.37	(1.68, 3.34)	2.88	(2.19, 3.79)	8.27	(4.04, 16.91)	7.35	(3.96, 13.64)
Age 50-54	3.10	(2.44, 3.94)	3.78	(2.66, 5.36)	16.60	(10.80, 25.53)	9.77	(4.84, 19.72)
Age 55+	3.99	(3.09, 5.14)	4.07	(2.98, 5.56)	25.24	(12.84, 49.59)	14.51	(7.60, 27.69)
Sex (Female)	1.31	(1.27, 1.36)	1.26	(1.04, 1.54)	1.50	(1.42, 1.58)	1.05	(0.76, 1.44)
SES	0.53	(0.47, 0.60)	0.58	(0.48, 0.70)	0.48	(0.40, 0.58)	0.58	(0.42, 0.80)
BMI ≥ 30	1.14	(0.98, 1.32)	0.96	(0.72, 1.30)	1.39	(1.19, 1.62)	1.13	(0.72, 1.79)
Alcohol consumption	1.01	(1.00, 1.01)	1.00	(1.00, 1.01)	1.00	(0.99, 1.00)	1.01	(0.99, 1.02)
Physical inactivity	1.18	(1.13, 1.22)	1.13	(0.92, 1.39)	1.32	(1.18, 1.48)	0.99	(0.71, 1.39)
Asthma	1.47	(1.38, 1.56)	1.51	(1.04, 2.18)	1.90	(1.67, 2.17)	1.47	(0.80, 2.73)
Myocardial infarction	1.46	(1.11, 1.91)	0.85	(0.27, 2.63)	1.84	(1.22, 2.79)	3.49	(0.87, 14.09)
Angina pectoris	1.26	(0.94, 1.69)	1.62	(0.79, 3.32)	1.26	(1.02, 1.55)	0.61	(0.15, 2.45)
Cerebrovascular diseases	1.10	(1.04, 1.16)	1.65	(0.84, 3.22)	1.46	(1.28, 1.66)	1.79	(0.71, 4.50)
Migraine	1.16	(1.11, 1.21)	1.31	(1.05, 1.62)	1.13	(1.04, 1.22)	1.13	(0.79, 1.61)
Depression	1.81	(1.65, 1.99)	1.35	(1.06, 1.72)	2.13	(1.89, 2.40)	1.79	(1.24, 2.58)
Diabetes	1.57	(1.36, 1.82)	2.12	(1.35, 3.31)	1.47	(1.26, 1.72)	2.94	(1.64, 5.28)
Quitting smoking	0.89	(0.81, 0.98)	0.90	(0.72, 1.12)	0.92	(0.82, 1.04)	0.84	(0.58, 1.22)

Supplementary Table 3. Hazard ratios for effect modifiers

	FPS			
	Any work disability		Disability pension	
Quit*Age(35-39)	0.85	(0.48, 1.54)	0.48	(0.10, 2.39)
Quit*Age(40-44)	0.88	(0.81, 0.96)	0.16	(0.01, 2.60)
Quit*Age(45-49)	1.03	(0.60, 1.75)	0.49	(0.17, 1.47)
Quit*Age(50-54)	1.09	(0.71, 1.68)	0.50	(0.18, 1.39)
Quit*Age(55+)	1.31	(1.07, 1.60)	0.93	(0.27, 3.22)
Quit*BMI>30	0.90	(0.52, 1.53)	0.86	(0.74, 0.99)
Quit*Physical inactivity	0.82	(0.69, 0.98)	0.90	(0.70, 1.16)
Quit*Low SES	1.04	(0.82, 1.33)	0.83	(0.63, 1.09)
Quit*Sex (Female)	1.16	(0.99, 1.36)	0.86	(0.64, 1.15)
Quit*log(Follow-up time)	0.95	(0.91, 0.99)	- *	- *
	HeSSup			
	Any work disability		Disability pension	
Quit*Age(35-39)	1.14	(0.54, 2.39)	1.15	(0.14, 9.59)
Quit*Age(40-44)	0.99	(0.37, 2.62)	2.69	(0.29, 25.14)
Quit*Age(45-49)	0.89	(0.47, 1.69)	1.89	(0.37, 9.70)
Quit*Age(50-54)	1.20	(0.53, 2.75)	3.10	(0.52, 18.47)
Quit*Age(55+)	1.28	(0.63, 2.59)	1.98	(0.37, 10.64)
Quit*BMI>30	1.51	(0.74, 3.10)	0.76	(0.21, 2.80)
Quit*Physical inactivity	0.77	(0.46, 1.28)	1.28	(0.36, 1.98)
Quit*Low SES	0.68	(0.42, 1.10)	0.89	(0.40, 2.00)
Quit*Sex (Female)	1.01	(0.65, 1.59)	1.27	(0.59, 2.75)
Quit*log(Follow-up time)	0.94	(0.69, 1.28)	- *	- *

*Hazard ratio could not be reliably estimated
